# Nucleosome Positioning with Set of Key Positions and Nucleosome Affinity

**DOI:** 10.2174/1874120701408010166

**Published:** 2014-12-31

**Authors:** Jia Wang, Shuai Liu, Weina Fu

**Affiliations:** 1 Experimental Instrument Center, Dalian Polytechnic University, Dalian, Liaoning, 116034, China;; 2 College of Computer Science, Inner Mongolia University, Hohhot, Inner Mongolia, 010012, China; 3 School of Physical Science and Technology, Inner Mongolia University, Inner Mongolia, 010012, China

**Keywords:** Affinity, DNA sequence, key position, neural network, nucleosome positioning

## Abstract

The formation and precise positioning of nucleosome in chromatin occupies a very important role in studying life process. Today, there are many researchers who discovered that the positioning where the location of a DNA sequence fragment wraps around a histone octamer in genome is not random but regular. However, the positioning is closely relevant to the concrete sequence of core DNA. So in this paper, we analyzed the relation between the affinity and sequence structure of core DNA, and extracted the set of key positions. In these positions, the nucleotide sequences probably occupy mainly action in the binding. First, we simplified and formatted the experimental data with the affinity. Then, to find the key positions in the wrapping, we used neural network to analyze the positive and negative effects of nucleosome generation for each position in core DNA sequences. However, we reached a class of weights with every position to describe this effect. Finally, based on the positions with high weights, we analyzed the reason why the chosen positions are key positions, and used these positions to construct a model for nucleosome positioning prediction. Experimental results show the effectiveness of our method.

## 1. INTRODUCTION

Admittedly, nucleosome is the basic structural unit of chromatin and is constructed by a DNA fragment (core DNA) and a histone octamer. Usually, the length of DNA around histone octamer is about 147 basepair, and wrapped over a histone octamer about 1.65 circles. Though the core DNA length is different for different organisms, based on cell type and areas of chromatin, it is known that nucleosome occupies 75% - 90% of genome, which means that nucleosome plays a role in life process. In fact, researchers find that nucleosome positioning plays a role in transcription regulation, gene expression and splicing [1]. However, not all base-pairs function equally in histone octamer wrapped. Meantime, different kinds of histone octamer show different preference to DNA fragment showing that nucleosome has its DNA sequence preference [2-4].

Earlier, Kornberg first presented nucleosome positioning based on statistics with barrier model [5]. He found that the nucleosome positioning is highly certain. In recent years,  the statistical model was under brisk research. Yuan and  Mavrich *et al.* researched in the statistical model and found the nucleosome positioning obeyed statistics outside barrier because of the electrostatic and steric hindrance effects [6-8]. They found that determinacy of nucleosome is lower when the position is farther from the barrier. Then, Fu and Schones studied in nucleosome positioning by human genome, and supported viewpoint of statistical positioning through analysis of the difference in yeast and human cells [9, 10]. Zhang and Stein also found that the DNA sequence preference is determined mainly in rotating position of nucleosome, but limited in translational displacements [11, 12]. Ioshikhes counted and computed standard distribution of AA/TT in core DNA sequence [13]. Leimgruber further compared distributional correlation of diad AA/TT both in experimental DNA sequence and standard distribution. He found that lack of nucleosome corresponds with the valley region of associated curve, and center of nucleosome corresponds with the peak region of associated curve [14].

In recent years, there were more predicting models presented with the appearance of *in vivo* nucleosome positioning data sample [15, 16]. Zhao *et al.* studied classified nucleosome preference and repellence sequences of yeast, drosophila and human by applied diversity of increment [17]. Their high level accuracy supported the viewpoint of DNA sequence positioning. Liu *et al.* used curvature profile model to predict properties of nucleosome positioning at target sites of TSS, TFBS, SNP and miRNA [18]. Recently, Becker *et al.* presented a variable optimal statistical model in nucleosome positioning [19]. This model used a study-predict method to predict probability distribution of nucleosome.

Since Segal and coworkers concluded that the affinity of DNA sequence fragment and histone octamer determines whether a DNA sequence fragment can wrap around a histone octamer. They experimented *in vivo* and *in vitro* with DNA sequence fragment and histone octamer of chicken [2]. Later, their viewpoint was extended by Field and Kaplan [3-4].

So, in this paper, we used both the affinity and flexibility approaches to improve our research. First, we present our material and methods in this paper. Then, we show our experimental results and discussion. Finally, we conclude our research.

(1) $$w_{i}=\left\{\begin{array}{c} \frac{{\mathrm{af}}_{i}}{n_{1i}-n_{\mathrm{2i}}},{\mathrm{af}}_{i}\geq 0\\ \frac{{\mathrm{af}}_{i}}{n_{\mathrm{2i}}-n_{1i}},{\mathrm{af}}_{i}<0 \end{array}\right.$$

## 2. MATERIAL AND METHODS

Open data in this paper is taken from experimental results of Kaplan *et al.* in [20], which is available on website (http://genie.weizmann.ac.il/pubs/nucleosomes08/nucleosomes08_data.html). In the data of synthetic oligonucleotides, we preferred synthetic oligonucleotides measured by microarray to the ones measured by sequencing because microarray has higher accuracy. All data are created in a pool of ~40,000 double-stranded oligonucleotides of length 150bp, and each combined with limited amounts of chicken histone octamers. Then, the wrapped ones are extracted that had successfully competed to form nucleosomes. Finally, the affinity is calculated as the log-ratio between the reconstituted fraction and the initial pool as a measure. The results are calculated by oligonucleotides that were sequenced at least once and at most 500 times in each experiment.

Amount of Data used is 43796 in this paper, which contains 25108 ‘positive’ sequence fragments (affinities of these sequences are positive) and 18688 ‘negative’ sequence fragments (affinities of these sequences are negative). We assume that the positive sequences are those who have higher probability to combine the histone octamer, and the negative ones have lower probability to form nucleosome. Then, in order to winnow the data with lower properties, we choose the data with affinity more than 1 or less than 0. Then, we have 11539 positive sequences and 5221 negative sequences.

We first count the frequency of each diad in the data. The results are presented in Fig. (**1**), where left one is frequency of diad in all data, middle one is frequency in positive data and right one is frequency in negative data. We found that diad CC/CG/GC/GG occupies high ratio in core-DNA with positive affinity and AA, AT, TA, TT occupy high ratio in core-DNA with negative affinity [21-23]. This confirms previous observations [24]. We also have that diad AC shows high ratio in core-DNA with positive affinity.

**Fig. (1):**
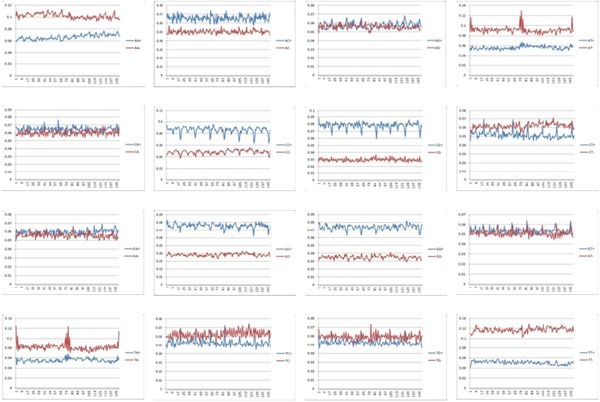
Frequency of diad in experimental data, the sub-figures are from AA at upper left to TT at lower right sequencing with ACGT, the blue line in each sub-figure shows the diad in core-DNA with positive affinity and the red line shows the diad in core-DNA with negative affinity.

Then, we calculate w_i_ for each DNA sequence fragment i in Eq.1, where n1i denotes the number of positive diad and n2i denotes the number of negative diad. Then, we divide all data into 10 parts equally and use neural network and leave-one-out method to process (only for study). In this case, we get the weight result for every position. Moreover, we have three experimental results to validate our conclusions, where the only difference between them is the different chosen diad. We reach the mixed result in the following section.

## 3. RESULTS AND DISCUSSION

Why AA/AT and CC/CG appeared frequently in core-DNA? We assume that it is because of the physical form of the diad structure. In a suitable position, it helps core-DNA to wrap at the histone octamer. This paper chooses *in vitro* data from Kaplan because *in vitro* has least disturbance than *in vivo*.

First, we used the training data to reach the key position in these core-DNAs for conducting three experiments. These three experiments are different from each other only in their diad. In the first experiment, we use diad CC/CG/GC/GG as positive training dataset and diad AA/AT/TA/TT as negative training dataset. In the second experiment, we dropped diad TA in the negative dataset because the frequency of diad TA does not show significant differences between positive and negative datasets. In the third experiment, we added diad AC as a positive comparison because the diad AC also shows significant differences between positive and negative datasets. Then, the training results of these three experiments, which are the training weights of all positions, are shown in Fig. (**2**).

**Fig. (2):**
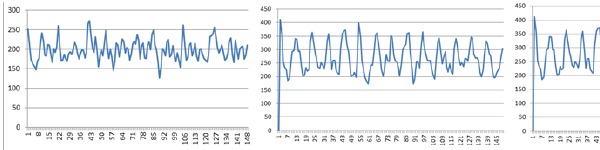
Training weights of all positions for the three experiments, the left sub-figure shows the result with the first experiment (CC/CG/GC/GG and AA/AT/TA/TT), the middle sub-figure shows the result with the second experiment (CC/CG/GC/GG and AA/AT/TT), the right sub-figure shows the result with the third experiment (AC/CC/CG/GC/ GG and AA/AT/TA/TT).

After that, we showed our experimental results in Table **1**, where the only difference between them is the chosen diad. To make the paper simple, we rename them to 4-4, 4-3 and 5-4 instead. The Accuracy (Acc), Sensitiveness (Sen), positive predictive value (PPV) and Matthews’s correlation coefficient (MCC) denote the 4 most important indexes in the experiment. The formulas of these 4 indexes are presented in Eqs.2-5, where true positive (TP), false positive (FP), true negative (TN), and false negative (FN) are all known in these experiments. In the first experiment, we have TP=786.3, FP=367.5, TN=1763.1 and FN=105.6 (mean of ten times with 9-1 model). In the second experiment, we have TP=852.6, FP=301.2, TN=1678.2 and FN=190.5. In the third experiment, we have TP=859.8, FP=294, TN=1673.7 and FN=195.0.

**Table 1:** Results of experiment.

**Chosen diad**	**The best positions (with largest weights)**	**Key indexes**			
		**Acc**	**Sen**	**PPV**	**MCC**
4-4	1,22,42,43,44,53,65,85,86, 95	84.3	88.2	68.1	66.6
4-3	2,13,14,37,45,54,75,105, 106,132	83.7	81.7	73.9	65.1
5-4	1,53,54,65,66,75,76,118, 127,131	83.8	81.5	74.5	65.3

In Table **1**, we find that the PPV and MCC are not large enough. This means that our method is not suitable for application on a positive sample. In other words, it is probably true when we test a sample as negative, but it is not so credible when we test a sample as positive.

But as a basic research, this paper presents a new method to predict nucleosome positioning with only key positions. In other words, the focus is on those positions that lead to the winding of core-DNA around histone octamer. Therefore, we are not concerned about the prediction rate here because this test only uses 10 or 20 direct factors without using a more complex computation which would require more features to be computed from the direct factors such as ones we applied.

(2) $$\mathrm{Acc}=\frac{\mathrm{TP}+\mathrm{TN}}{\mathrm{TP}+\mathrm{FP}+\mathrm{TN}+\mathrm{FN}}$$

(3) $$\mathrm{Sen}=\frac{\mathrm{TP}}{\mathrm{TP}+\mathrm{FN}}$$

(4) $$\mathrm{PPV}=\frac{\mathrm{TP}}{\mathrm{TP}+\mathrm{FP}}$$

From Table **1**, we found that the best positions’ distribution of the 3 experiments is equal. This can also be seen in Fig. (**2**) (peaks in each subfigure). In Table ****1****, we found that position weights in 4-4 experiment shake more frequently than the other two experiments. But we can also observe that the distribution of peaks shows a similar period. When we added the positions with largest weights to 20 in Table ****2****, we found that they are similar to each other in some properties. So, we believe that these properties may be found in other species. On the other hand, though our results with chicken did not come up with good results than others’ with yeast, yet we can conclude that genome in chicken is more complex than in yeast.

(5) $$\mathrm{MCC}=\frac{\mathrm{TP}\cdot \mathrm{TN}-\mathrm{FP}\cdot \mathrm{FN}}{\sqrt{\left(\mathrm{TP}+\mathrm{FN})\cdot (\mathrm{TP}+\mathrm{FP})\cdot (\mathrm{TN}+\mathrm{FN})\cdot (\mathrm{TN}+\mathrm{FP}\right)}}$$

**Table 2:** 20 Key Positions Used in Experiments.

4-4	1,2,22,33,42,43,44,53,54,64,65,74,75,85,86, 95,106,117,127,128
4-3	2,13,14,37,45,46,54,65,66,75,84,86,91,105, 106,113,117,118,121,132
5-4	1,2,11,12,22,43,46,53,54,55,56,66,75,76,85, 86,87,96,117,127

Since the histone octamer in chicken, yeast and other eukaryotes is structurally similar, in physical thinking, core-associated DNA should also be similar. So, the differences should be localized somewhere else and this will lead to further specialized research in the field. Our research suggests a brand new idea that only key positions in core-DNA are similar in these organisms, other sequences in core-DNA may be involved in other regulative chromatin functions such as DNA methylation and histone modification in living beings, because they are the nearest part of them. So, we think that our results are meaningful.

**Fig. (3):**
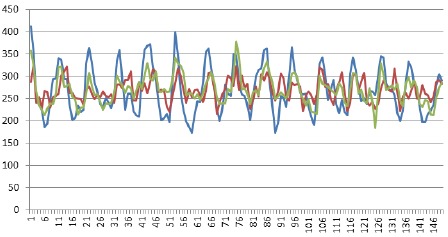
Comparison of the training weights in all positions for the three experiments, the blue line is for 4-4, red line in for 4-3 and green line is for 5-4.

**Fig. (4):**
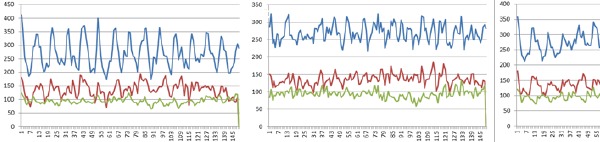
Comparison of the training weights at all positions for the three datasets in each experiment, the left sub-figure is for 4-4, the middle sub-figure is for 4-3, the right sub-figure is for 5-4, in each sub-figure, the blue line is for positive + negative dataset, red line in only for positive dataset, 4-3 and green line is for negative dataset.

Finally, we compared the weights of all positions found in all these three experiments (Fig. **3**). Also, we used only positive and negative datasets in the three experiments to compute the weights of positions (Fig. **4**). (Figs. **3** & **4**), show that all these three experiments reach similar weights of positions, and the weights of positions are also similar between the three datasets we have chosen. For example, Fig. (3) shows that the tendency and extreme points are all similar between the three lines. However, Fig. (4) shows that the maximum points in each sub-figure are similar between the blue and red lines, and the minimum points in each sub-figure are similar between the blue and green lines. This means that the key positions exist as a natural property of core-DNA.

## CONCLUSION

In this paper, we presented a novel method to predict nucleosome positioning. In this method, we used a novel thinking involving both key positions and affinity. We used Segal’s data to find the key positions and used these positions only to predict if a DNA sequence fragment is a core-DNA. Experimental results showed its effectiveness.

Next, we divided each DNA sequence fragment into three sub-sequences. This is because the combined positions in core-DNA not only have a high frequency to appear in the middle sub-sequence, but also have a relative low frequency in the first and third sub-sequences. So, we divided each DNA sequence fragment (150bp) to front (1-30), mid (31-120) and last (121-150), assume that these sub-sequences have different structures and distribution of bases. We plan to investigate this further in our next study whether there are better results.

## References

[1] Hui L., Zi-Heng Z., Ji-Hong G., Shui-Geng Z. (2012). Transcriptional regulation functions of nucleosome positioning: A survey.. Prog. Biochem. Biophy..

[2] Segal E., Fondufe-Mittendorf Y. (2006). Chen. L, A. Thåström, Y. Field, I. K. Moore, J. P. Wang and J. Widom, “A genomic code for nucleosome positioning.. Nature.

[3] Field Y., Kaplan N., Fondufe-Mittendorf Y., Moore I.K., Sharon E., Lubling Y., Widom J., Segal E. (2008). Distinct modes of regulation by chromatin encoded through nucleosome positioning signals.. PLOS Comput. Biol..

[4] Kaplan N., Moore I.K., Fondufe-Mittendorf Y., Gossett A.J., Tillo D., Field Y., LeProust E.M., Hughes T.R., Lieb J.D., Widom J., Segal E. (2009). The DNA-encoded nucleosome organization of a eukaryotic genome.. Nature.

[5] Kornberg R.D., Stryer L. (1988). Statistical distributions of nucleosomes: Nonrandom locations by a stochastic mechanism.. Nucleic Acids Res..

[6] Yuan G.C., Liu Y.J., Dion M.F., Slack M.D., Wu L.F., Altschuler S.J., Rando O.J. (2005). Genome-scale identification of nucleosome positions in S. cerevisiae.. Science.

[7] Mavrich T.N., Ioshikhes I.P., Venters B.J., Jiang C., Tomsho L.P., Qi J., Schuster S.C., Albert I., Pugh B.F. (2008). A barrier nucleosome model for statistical positioning of nucleosomes throughout the yeast genome.. Genome Res..

[8] Mavrich T.N., Jiang C., Ioshikhes I.P., Li X., Venters B.J., Zanton S.J., Tomsho L.P., Qi J., Glaser R.L., Schuster S.C., Gilmour D.S., Albert I., Pugh B.F. (2008). Nucleosome organization in the Drosophila genome.. Nature.

[9] Fu Y., Sinha M., Peterson C.L., Weng Z. (2008). The insulator binding protein CTCF positions 20 nucleosomes around its binding sites across the human genome.. PLoS Genet..

[10] Schones D.E., Cui K., Cuddapah S., Roh T.Y., Barski A., Wang Z., Wei G., Zhao K. (2008). Dynamic regulation of nucleosome positioning in the human genome.. Cell.

[11] Zhang Y., Moqtaderi Z., Rattner B.P., Euskirchen G., Snyder M., Kadonaga J.T., Liu X.S., Struhl K. (2009). Intrinsic histone-DNA interactions are not the major determinant of nucleosome positions *in vivo*.. Nat. Struct. Mol. Biol..

[12] Stein A., Takasuka T.E., Collings C.K. (2009). Are nucleosome positions *in vivo* primarily determined by histone-DNA sequence preferences?. Nucleic Acids Res..

[13] Ioshikhes I.P., Albert I., Zanton S.J., Pugh B.F. (2006). Nueleosome positions predicted through comparative genomics.. Nat. Genet..

[14] Leimgruber E., Seguin-Estevez Q. (2009). Nucleosome eviction from MHC class promoters controls position of the transcription start site.. Nucleic Acids Res..

[15] Morris R.T. (2010). T. R. O′Connor and J. J. Wyrick, “Ceres: Software for the integrated analysis of transcription factor binding sites and nucleosome positions in Saccharomyces cerevisiae.. Bioinformatics.

[16] Yi X., Cai Y.D., He Z. (2010). C. WeiRen and K. Xiangyin, “Prediction of nucleosome positioning based on transcription factor binding sites.. PLoS One.

[17] Zhao X., Pei Z., Liu J., Qin S., Cai L. (2010). Prediction of nucleosome DNA formation potential and nucleosome positioning using increment of diversity combined with quadratic discriminant analysis.. Chrom. Res..

[18] Liu H., Duan X., Yu S., Sun X. (2011). Analysis of nucleosome positioning determined by DNA helix curvature in the human genome.. BMC Genome.

[19] Becker J., Yau C., Hancock J.M., Holmes C.C. (2013). NucleoFinder: A statistical approach for the detection of nucleosome positions.. Bioinformatics.

[20] Kaplan N., Moore I.K., Fondufe-Mittendorf Y., Gossett A.J., Tillo D., Field Y., LeProust E.M., Hughes T.R., Lieb J.D., Widom J., Segal E. (2009). The DNA-encoded nucleosome organization of a eukaryotic genome.. Nature.

[21] Satchwell S.C., Drew H.R., Travers A.A. (1986). Sequence periodicities in chicken nucleosome core DNA.. J. Mol. Biol..

[22] Negri R., Buttinelli M., Panetta G., De Arcangelis V., Di Mauro E., Travers A. (2001). Sequence dependence of translational positioning of core nucleosomes.. J. Mol. Biol..

[23] Bolshoy A., Shapiro K., Trifonov E.N., Ioshikhes I. (1997). Enhancement of the nucleosomal pattern in sequences of lower complexity.. Nucleic Acids Res..

[24] Caserta M., Agricola E., Churcher M., Hiriart E., Verdone L., Di Mauro E., Travers A. (2009). A translational signature for nucleosome positioning *in vivo*.. Nucleic Acids Res..

